# Intravenous branched-chain amino-acid-free solution for the treatment of metabolic decompensation episodes in Spanish pediatric patients with maple syrup urine disease

**DOI:** 10.3389/fped.2022.969741

**Published:** 2022-08-15

**Authors:** Paula Sánchez-Pintos, Silvia Meavilla, María Goretti López-Ramos, Ángeles García-Cazorla, Maria L. Couce

**Affiliations:** ^1^Diagnosis and Treatment Unit of Congenital Metabolic Diseases, Department of Paediatrics, University Clinical Hospital of Santiago de Compostela, Santiago de Compostela, Spain; ^2^IDIS-Health Research Institute of Santiago de Compostela, Santiago de Compostela, Spain; ^3^CIBERER, Instituto Salud Carlos III, Madrid, Spain; ^4^MetabERN, European Reference Network for Rare Hereditary Metabolic Disorders, Udine, Italy; ^5^Metabolic Diseases Unit, Neurology Department, CIBERER and MetabERN, Sant Joan de Déu Hospital, Barcelona, Spain

**Keywords:** dietary treatment, intravenous branched-chain amino acid-free formula, metabolic decompensation, metabolic emergency treatment, MSUD

## Abstract

**Background:**

Metabolic decompensation episodes (DEs) in Maple Syrup urine disease (MSUD) result in brain accumulation of toxic branched-chain amino acids (BCAAs) and their respective branched-chain α-keto acids that could induce neuroinflammation, disturb brain bioenergetics, and alter glutamate and glutamine synthesis. These episodes require immediate intervention to prevent irreversible neurological damage. Intravenous (IV) administration of BCAA-free solution could represent a powerful alternative for emergency treatment of decompensations.

**Methods:**

This pediatric series discusses the management of DEs in MSUD patients with IV BCAA-free solution, as an emergency treatment for DEs or as a prophylactic in cases requiring surgery. Clinical evolution, amino acid profile and adverse effects were evaluated.

**Results:**

We evaluated the use of BCAA-free solution in 5 DEs in 5 MSUD pediatric patients, all with significantly elevated plasma leucine levels at admission (699–3296 μmol/L) and in 1 episode of risk of DE due to surgery. Leucine normalization was achieved in all cases with resolution or improvement of clinical symptoms following IV BCAA-free solution. The duration of administration ranged from 3–20 days. Administration of IV BCAA-free solution at the beginning of a DE could reverse depletion of the amino acids that compete with BCAAs for the LAT1 transporter, and the observed depletion of alanine, despite IV alanine supplementation. No related adverse events were observed.

**Conclusions:**

Administration of standardized IV BCAA-free solution in emergency settings constitutes an important and safe alternative for the treatment of DEs in MSUD, especially in pediatric patients for whom oral or enteral treatment is not viable.

## Introduction

Maple syrup urine disease (MSUD, OMIM 248600) is a rare inborn metabolic disorder involving a deficiency of the branched chain α-ketoacid dehydrogenase complex that results in tissue accumulation of the branched-chain amino acids (BCAAs) and their respective branched-chain α-keto acids (BCKA): α-ketoisocaproic (αKIC), α-ketoisovaleric and α-keto-β-methylvaleric acids. The elevated blood level of BCAAs impairs cerebral uptake of large neutral amino acids thought the blood-brain barrier, decreasing their concentration in brain (1). In the pathogenesis of neurological damage in MSUD, an increasingly role of the neurotoxic properties of the BCAA and BCKA is recognized (1), disturbing brain bioenergetics and redox homeostasis (2), inducing neuroinflammation (3) and contributing to a reduction of brain glutamate, glutamine, and gamma-aminobutyrate concentrations (4). Leucine and KIC are considered the main neurotoxins in this disorder. Both act as metabolic inhibitors and αKIC, which enters the brain *via* the monocarboxylate transporter (SLC16A1), also act as an uncoupler of oxidative phosphorylation (4). The increase of αKIC in tissues explains the inverse relationship of leucine to glutamate, glutamine, and alanine in classical MSUD patients as it reverses normal flow through branched-chain amino acid transaminase (BCAT1), depletes tissues of glutamate and indirectly drives flux through glutamate-pyruvate transaminase (5). The standard therapeutic approach includes BCAA restriction by natural dietary protein limitation and supplementation of BCAA-free formula. Treatment for severe forms may involve liver transplantation to replace functional branched-chain α-ketoacid dehydrogenase complex in the liver. There are stills unknowns about the treatment outcomes and the risk of decompensation episodes (DEs), usually precipitated by metabolic stress circumstances like infection (6, 7), persists despite chronic dietary treatment adherence. In newborns, DEs generally present as irritability, vomiting, lethargy, altered consciousness, apnoea, coma and opisthotonos, and in older patients, symptoms may include anorexia, vomiting, muscle fatigue, altered level of consciousness, coma, psychiatric symptoms, movement disorders and ataxia (8). During metabolic DE, there is a rapid accumulation of toxic BCAAs, mostly leucine, resulting in damage to neurological tissue (9, 10) due to a disturbance of the brain aerobic metabolism by compromising the citric acid cycle and the electron flow through the respiratory chain. Immediate intervention and treatment are essential to avoid irreversible neurological impairment (8, 11).

The goal in acute management is to suppress catabolism and promote protein anabolism. In the absence of standardized approved treatments, the common approach for managing DE generally involves increasing the BCAA-free amino acid formula, usually *via* a nasogastric tube (7, 12), decreasing or stopping leucine intake, supplementing valine and isoleucine (20–120mg/kg/day), providing sufficient caloric and fluid intake to guarantee anabolism (1.5–3-times estimated energy expenditure (5, 13). In instances where this treatment is inadequate or insufficiently effective, especially in the pediatric population, or when leucine plasma levels are critically elevated and associated with severe clinical symptoms, extracorporeal procedures, including haemodialysis (HD) and/or haemofiltration (HF) are necessary to prevent neurotoxicity and death (7, 8, 12). However, as extracorporeal techniques may cause infection, increased catabolism and require an appropriately equipped facility and experienced personnel, the management of DE with non-invasive effective nutritional interventions is an attractive option, and has been shown to be effective in previous cases (14). Publications on the Japanese cohort of MSUD patients reported that two patients had been treated with intravenous (IV) “hyperalimentation” with good results, although the details of this treatment are not clear (15, 16). That said, IV BCAA-free formulas are not readily available in many countries, including in Spain. Given the rarity of this disorder, and the scarcity of clinical studies comparing treatments, we report here on 5 pediatric and adolescent MSUD patients experiencing metabolic decompensation at two reference centers for metabolic diseases in Spain who were treated with an IV BCAA-free solution and discuss the results in the context of a review of the available literature on the subject.

## Patients and methods

We developed a retrospective observational study of pediatric MSUD patients hospitalized at two tertiary Spanish Reference Centers for Congenital Metabolic Diseases during the period 2012–2020. Information was collected from clinical records. Informed consent was obtained of the parents.

All patients were treated with the same IV BCAA-free solution, a 5% amino acid solution whose formulation is detailed in Supplementary Table 1. The study variables include age, gender, method of MSUD diagnosis (newborn screening/clinical diagnosis), basal treatment, precipitating factors for DE (neonatal onset/infection/vaccination/surgery/other type of metabolic stress/lack of dietary treatment adherence), clinical symptoms at hospital entrance, prior and secondary neurologic involvement, plasma leucine levels (at admission, its evolution during the DE and at discharge), BCAA-free IV solution (dose, duration, indication to its use, and related adverse drug reactions and adverse events), other therapeutic modalities employed, duration of hospitalization and clinical situation at discharge.

Plasma leucine levels were determined by tandem mass spectrometry in dried-blood samples. Amino acid analyses from dried blood spot samples include a preparative step of elution and deproteinisation with 3% trichloroacetic acid. The analysis was carried out by ion-exchange chromatography after deproteinisation of the sample with 5-sulfosalicylic acid and a post-column reaction with ninhydrin.

## Results

Our series consisted of 5 cases of metabolic DE in 5 MSUD pediatric patients (aged 1 week to 16 years) that required hospital admission, and 1 case of risk of DE due to fasting for intestinal surgery (patient 1). DE was defined as plasma leucine levels >381 μmol/L (5.0 mg/dL) at hospital admission. All patients were diagnosed by newborn screening. Patient characteristics are summarized in Table 1. The series include 2 cases of DE associated with neonatal clinical onset (1 complicated with intestinal perforation), 3 cases of DE with concomitant infection and vomiting, and 1 case of risk of DE due to surgery (in patient 1, who had a history of intestinal perforation). Clinical management of the DE followed international (5, 7) and Spanish treatment guidelines (17), which recommend treating the precipitating factor and stopping leucine intake, while providing sufficient calories, insulin, BCAA-free amino acids solutions, isoleucine, and valine to achieve sustained net protein synthesis in tissues. Upon admission, all patients were provided with a high IV caloric intake consisting of carbohydrates and lipids. Protein intake (parenteral +/- enteral) ranged from 2–3.5 g/kg/day as BCAA-free amino acids and isoleucine and valine supplementation was increased (20– 120 mg/kg/day) with titration of plasma concentrations. In addition, three patients (cases 1, 3 and 4) were treated with HD/HF and measures to maintain normal serum sodium and prevent or reverse cerebral oedema were establish (hypertonic saline, furosemide and in case 4 mannitol).

**Table 1:** Patient characteristics at admission.

**Center**	**Patient**	**Case**	**Admission date**	**Age at admission**	**Trigger**	**Signs and symptoms at admission**
1	1 female	1	02/2020	1 week	Neonatal	Neurological disorders (boxing movements)
		2	07/2020	5 months	Surgery	None (previous neurological injury on MRI)
2	2 male	3	02/2016	7 days	Neonatal	Irritability; coma with cerebral oedema 12 h after admission
	3 male	4	05/2015	2 years 3 months	Infection	Vomiting, nausea, respiratory distress
	4 male	5	12/2011	9 years	Infection	Vomiting, nausea, fever
	5 male	6	02/2017	16 years	Infection	Vomiting, nausea

Three patients (patients 1–3) initially received only IV BCAA-free solution and 2 (patients 4 and 5) received a combined IV + enteral BCAA-free formulation. IV BCAA-free solution was administered twice in patient 1, for whom oral feeding was impossible due to intestinal perforation, in 3 patients (patients 3–5) for oral feeding intolerance with vomiting due to infection, and in the remaining patient (patient 2), for whom oral feeding was delayed due to poor enteral tolerance and frequent vomiting despite good metabolic control. Details and treatments for each episode are provided in Table 2, while the evolution of leucine levels is presented in Supplementary Figure 1. In all episodes, except case 2 (patient 1) in which BCAA-free solution was administered preventively, leucine level at admission was above the decompensation threshold (669–3296 μmol/L).

**Table 2:** Leucine plasma levels, treatments, and outcomes.

**Patient**	**Case**	**Reason for treatment**	**Leucine levels (**μ**mol/L)**			**IV BCAA-free treatment duration (days)**	**IV BCAA-free dose (g/kg/day)**	**Hospital ization (days)**	**Concomitant treatments during DE**	**Clinical notes**
			**Admission**	**End of IV BCAA-free treatment**	**Discharge**					
1	1	High leucine Plasma level, enteral feeding not possible	3296	199	38	8	2.75	30	– IV dextrose and lipids - Isoleucine - Valine - Thiamine - Alanine - HD/HF - Hypertonic saline and furosemide	Intestinal perforation before IV BCAA-free treatment. Perforation was resolved at discharge, with no sequelae.
	2	High leucine plasma level, surgery (2nd admission)	167	29	82	7	2.75	11	- IV dextrose and lipids - Isoleucine - Valine - Thiamine - Alanine	Metabolic acidosis prior to IV BCAA-free solution, which resolved at discharge, with no ongoing sequelae.
2	3	High leucine level	2218	198	236	3	0.5-1	42	- IV dextrose and lipids – Isoleucine - Valine - Thiamine - Alanine - HD/HF - Hypertonic saline and furosemide	Hospital discharge was delayed because of poor enteral tolerance and frequent vomiting despite good metabolic control; no ongoing sequelae at discharge.
3	4	Clinical symptoms	699	46	23	20	1.5	65	- IV dextrose and lipids - Isoleucine - Valine - Thiamine - Alanine - HD/HF. - Hypertonic saline and furosemide - Mannitol.	Persistent oral intolerance after DE with frequent vomiting, forcing repeated stopping of enteral feeding. Discharged on physician's recommendation with ongoing sequelae: slow normalization of neurological symptoms.
4	5	Clinical symptoms and high leucine levels	938	320	282	7	0.5	13	- IV dextrose and lipids - Isoleucine - Valine Thiamine - Alanine	Resolution of symptoms at discharge and no ongoing sequelae.
5	6	High leucine plasma levels, vomiting	953	343	175	3	0.7	14	- IV dextrose and lipids Isoleucine - Valine -Thiamine - Alanine	Resolution of symptoms at discharge and no ongoing sequelae.

*HD/HF, haemodialysis/haemofiltration*.

The BCAA-free solution dosages employed over 24 h were 2–3 g/kg body weight for neonates and infants (<2 years) and 1–2 g/kg body weight for children (>2 years) and adolescents, except for patients 5 and 6 who received a lower dose ( ≤ 1 g/kg/day) due to rapid clinical improvement and resolution of vomiting that allowed combined treatment with oral and IV BCAA-free solutions. Treatment duration ranged from 3–20 days. Before 2016, BCAA-free solution was obtained from France for compassionate use, and subsequently was manufactured in Spain by local compounding pharmacies following the French composition.

The duration of BCAA-free IV treatment ranged from 3–8 days, except in patient 3, who experienced a severe metabolic encephalopathy episode 12 h after admission, with decreased level of consciousness and coma due to cerebral oedema related to adenovirus infection (concomitant leucine levels: 935 μmol/L). BCAA-free IV solution was not available at admission and was administered immediately after the onset of this episode, together with HF (duration, 12 h). Leucine level decreased to 563 μmol/L in 24 h, and subsequently decreased further to reach normal levels. Enteral intake of BCAA-free products was started 48 h after admission, but was discontinued due to persistent oral intolerance with frequent vomiting, despite transpyloric tube feeding. IV administration of BCAA-free solution was maintained.

In 4 of the described episodes a complete amino acid profile was obtained after beginning emergency treatment and before administration of IV BCAA-free solution. Depletion of 4 of the 7 amino acids that compete with BCAAs for the LAT1 transporter was observed (mean [range]: tyrosine (Tyr), 25.45 [34–84] μmol/L; histidine (His), 43.3 [34–106] μmol/L; methionine (Met), 7.8 [4.5–27] μmol/L; glutamine (Gln), 317 [303–709] μmol/L. Normal ranges in the local laboratory for neonates and children, respectively, are as follows: Tyr, 31–115 μmol/L and 34–82 μmol/L; His, 55–115 μmol/L and 54–106 μmol/L; Met: 19–51 and 11–27 μmol/L; Gln, 368–652 μmol/L and 373–709 μmol/L.

Alanine depletion was also observed despite regular IV alanine supplementation during decompensations (mean [range] 99.5 [97–103 μmol/L]; normal range, 158–314 μmol/L for neonates and 182–378 μmol/L for children). The amino acid profiles normalized after the introduction of IV BCAA-free solution. In all cases, treatment with the IV BCAA-free solution was discontinued once plasma leucine concentration reached <381 μmol/L and symptom resolution or clinical improvement was achieved. All patients were discharged based on the physician's assessment that the episode was considered sufficiently improved or completely resolved.

## Discussion

A literature review of available publications that mentioned the use of intravenous (IV) BCAA-free solution specifically in pediatric MSUD patients was carried out, the results of which are shown in Table 3. In a very limited number of individual case studies in the 1980s and 1990s IV BCAA-free solutions were used in neonates and children undergoing acute DEs with apparent effectiveness (18–20). In one of these early reports, Berry *et al*. reported that the value of IV BCAA-free solution lay in its simplicity of use (20). In the US one study reported on the treatment of 35 MSUD patients, 5 of whom had received an IV BCAA-free mixture (21). Again, the details of the mixture are unclear. One of the benefits reported with the BCAA-free solution is an improvement in the duration of the DE. More recently, a French cohort of MSUD patients has been reported on more extensively. This cohort, numbering more than 126 episodes from 54 patients (22), including an analysis of 17 episodes in 4 adult patients (23) and 30 episodes in pediatric patients (24) treated with IV BCAA-free solution; and a subsequent report on 35 patients, 20 of them pediatric (<16 years), 14 of whom received the IV BCAA-free solution (25). The most recent analysis of this cohort compared both adult and pediatric subgroups of patients treated with BCAA-free solution with those treated with conventional oral therapy (22). In this cohort the overall mean duration of hospitalization was 6.6 days with the oral/enteral BCAA-free formula and 5.4 days for the IV solution. The authors considered that, at least for adults, the IV formula tends to be associated with a shorter time to episode resolution. For the pediatric subgroup there was no difference between outcomes between groups. The safety and tolerability profile also appears to be favorable in all populations for both the oral/enteral and IV solution, with no serious adverse events reported (22).

**Table 3:** Reports of IV BCAA-free amino acid formula administration in pediatric MSUD patients.

**Author, Year**	**Patients, episodes**	**Treatment**	**Outcomes**	**Comments**
De Lonlay et al. (17)	126 episodes total: pediatric group (oral/enteral 65; IV 14)	Episodes treated with oral/enteral BCAA-free formula or IV BCAA-free solution	Oral/enteral vs. IV): percentage reaching normalization (83.1%, *n* = 54 vs. 85.7%, *n* = 12); mean (±SD) time to first leucine normalization (68.3 h [±53.4] vs. 84.1 h [±59.8]); and mean (±SD) time to episode resolution (8.8 days ( ± 6) vs. 6.8 days (±3.6), *p* = NS). The duration of hospitalization was the same in both treatment groups (mean 6.6 days)	Enteral or oral BCAA-free formula was frequently used in children (94%, adults: 44.4%) and the IV formulation was frequently used in adults (85.2%, children: 21.7%).
Abi-Wardé et al. (25)	20 pediatric patients out of 35 patient total	14 patients (including 6 <6 years) treated with IV BCAA-free solution (including adults)	Patients on oral BCAA spent 4.1 (1.4) days at the hospital, whereas IV BCAA patients spent 3.6 (1.3) days. Leucine level normalization was faster when patients were treated IV rather than orally (*p* = 0.015)	A clear comparison of IV vs. oral BCAA-free solution was not reported for the pediatric subset; haemodialysis was not excluded.
Alili et al. (19)	30 acute episodes in pediatric patients	IV BCAA free solution	At discharge, 82% (*n* = 18/22) of children and 84% (*n* = 67/80) of adults had a normalized leucine concentration	Haemodialysis in 4 children. Parenteral BCAA-free solution appeared effective and safe, providing an alternative to nasogastric route.
Morton et al. (16)	36 neonates, 5 received IV-PN	IV-PN: intravenous dextrose and amino acid mixture devoid of leucine, isoleucine, and valine	- Days until Leucine <400 μmol/L in IV-PN group (range): 3.5–8. - The average length of hospitalization in the series was 6 days (range 4–8 days).	- Asymptomatic at-risk infants were managed exclusively with oral feeding
Yoshino et al. (11)	Of 13 patients, 1 infant (2-year-old) received IV hyperalimentation	IV hyperalimentation	Leucine level reduction from 36.4 to 14.1 μmol/L in 3 days	Patient was also treated with haemodialysis.
Koga et al. (10)	8-year-old	IV hyperalimentation before, during and after surgery	Serum levels of BCAA increased 4.6- to 9.5-fold within 24 h after the operation but with no clinical symptoms of ketoacidotic attack nor any laboratory abnormality.	-
Berry et al. (15)	9 episodes in 5 pediatric patients	Regimen of modified parenteral nutrition used in 6 occasions (3 times with modified parenteral nutrition and 3 times with modified parents nutrition plus formula)	Effective reduction in leucine levels in all 6 episodes	Severe patients treated immediately with dialysis. Authors reported the value being the simplicity of use of the IV BCAA free formulation.
Thompson et al. (14)	1 newborn	IV amino-acid supplement with low concentration of BCAAs 6 h after haemofiltration	Control achieved within 8 h of haemofiltration	Major treatment was continuous venous haemofiltration.
Townsend et al. (13)	1 newborn	Parenteral nutrition mixture lacking leucine, isoleucine, and valine, supplementedwith *Intralipid* to provide an average of 125 kcal/kg/day	“Achieves and sustains BCAA removal with fewer risks than multiple exchange transfusions or peritoneal dialysis”	Total parenteral nutrition initiated after patient was unable to continue nasogastric feeding and peritoneal dialysis had failed due to the patient's hypotonia.

*BCAA, branched-chain amino acids; IV, intravenous; IV-PN, intravenous dextrose and BCAA-free amino acid mixture; NS, not significant*.

In this series, overall, the IV BCAA-free solution was administered for ≤ 8 days, which is in line with results from the pediatric subset from the French MSUD cohort (22). Treatment with the BCAA-free solution was associated with a normalization of leucine plasma levels and an improvement or resolution of symptoms in all cases. Such results are consistent with the outcomes that have been reported across studies using the BCAA-free mixture for the treatment of DE in MSUD patients (7, 18, 20–23, 25, 26). The average hospitalization in this series was biased by patient 3, whose hospital stay was prolonged due to ongoing motor sequalae and feeding intolerance. One area for potential improvement, as reflected by the experiences in this case series, would be the immediate availability of a BCAA-free solution as this would reduce the time to treatment, which in this series was delayed because it included the time for the pharmacies to process the request and to manufacture the product.

Since excessive leucine levels represent a clinical emergency, IV administration of BCAA-free formula may be a useful alternative to oral/enteral formulations in emergency settings if the patient is not able to receive, or cannot tolerate, oral treatment or enteral tubing (22). Moreover, in MSUD decompensation the accumulation of branched-chain ketoacids leads to a consumption of glutamate, aspartate, glutamine, and alanine, due to reverse transamination, that interferes with several critical biochemical processes, and contributes to energy failure and cerebral lactic acidosis (4). The BCAA-free formulation includes the seven amino acids (Tyr, His, Met, Gln, threonine, tryptofan, phenylalaline) that compete with BCAAs for entry into the brain *via* the LAT1 transporter, helping to reduce the cerebral damage induced by leucine. Therefore, as reflected in this case series, the early administration of IV BCAA-free protein solution can help to protect the brain against deficiencies of amino acids necessary for protein and neurotransmitter synthesis, and methyl group transfer, and minimizes the deleterious effects of metabolic decompensation in MSUD patients.

In the present case series, no adverse events were detected as associated with either the BCAA-free solution or with its administration. Again, this is consistent with the available literature, where no serious adverse drug reaction has been reported, (7, 18, 20–23, 25, 26), and this formulation has shown to be safe in both neonates and older children.

Extracorporeal leucine reduction by a variety of methods, including haemodialysis and/or hemofiltration (HD/HF) has been shown to be effective in managing excessively or stubbornly high leucine levels during metabolic decompensation (27). HD/HF is recommended because it achieves faster reductions in leucine levels that conservative treatment approaches. Over recent decades HD/HF have become a standard treatment for severe or treatment-resistant DE (7), together with the concomitant administration of BCAA-free formula, either administered orally or enterally (26, 28). Other publications in the following decades reported effective episode management using HD/HF combined with dietary BCAA restriction and calorie support in single pediatric cases (29–32). Of note, in 2016, in a report of 14 patients receiving HD/HF, the authors stated that in these cases IV BCAA-free solution was not available (33). Recently, a Turkish report involving 9 MSUD patients treated for 14 episodes with HD/HF and parenteral BCAA-free nutrition found this to be an effective treatment approach (12). Nevertheless, HD/HF is not without risk, such as infection, intestinal obstruction and increased catabolism. Moreover, HD/HF requires a high level of clinical expertise, particularly in very young infants. Thus, there have been longstanding efforts explore alternative approaches.

Traditionally, treatments to promote anabolism and to provide energy have been provided *via* enteral feeding tube (14, 17). However, a recent large French pediatric cohort (n=190) including 9 MSUD patients report a high degree of complications related with enteral tube feed for metabolic diseases, occurring in around 50% of cases. The most frequent complications described were difficulties for inserting the nasogastric tube, frequent removal of the tube by the patient and vomiting. These complications had a significant impact on both the child and on family's quality of life (34). Moreover, in certain cases the use of a nasogastric tube can be challenging to apply, such as when the patient is vomiting, is in coma or cannot tolerate it (23). In these circumstances the availability of an IV BCAA-free solution may offer a benefit over enteral administration.

Thus, there is a clear clinical need for alternatives to both HD/HF treatment and enteral BCAA-free formulas. Indeed, the IV BCAA-free formula has been used intermittently over the last 3 decades to this end. A case report from 1991 of 5 US patients being treated for 20 episodes explored the use of a parenteral BCAA-free solution, which successfully lowered leucine levels in their population (20). In the same year a report was published of a single patient in Australia who received IV supplementation in addition to HD/HF, although this formulation was not completely BCAA-free, it was proportionately very low in BCAAs (19). In the 1990s in a Japanese MSUD population of 13 patients, one patient was treated with both HD/HF and “IV hyperalimentation”(16). Since then, the French cohort of MSUD adult and pediatric patients has been treated on and off with IV BCAA solutions (22–25). The most comprehensive analysis of this population including a pediatric subpopulation of 79 episodes, compared outcomes for episodes treated with enteral BCAA-free and those who received IV BCAA (22). After excluding those patients who had received HD/HF, the percentage reaching normalization in enteral group was 83.1% (*n* = 54) compared with 85.7% (*n* = 12) for IV group. The duration of hospitalization per episode was 6.6 days for both groups, but median time to episode resolution was 7 days for enteral group and 5 days for IV group. However, these comparisons were limited by the small sample size of the pediatric IV group and the retrospective nature of the study. Nevertheless, those findings were suggestive of a clinical and, as the authors suggest, a practical benefit to using the IV solution in clinically challenging situations, such as when the patient is in a coma or cannot tolerate enteral feeding (22).

Availability of IV BCAA-free solution remains a key problem. Although shown to be safe and effective in various publications worldwide (14, 22, 35), in Spain and other countries IV BCAA-free solution (used in the above cases) is only available on demand in special circumstances. The solution was prepared by two local compounding pharmacies following the composition listed in Supplementary Table 1. In the US, a retrospective analysis of an American population of 184 MSUD patients collected over 3 decades reported that, “parenteral MSUD amino acid solutions are available from only a very limited number of specialty pharmacies, and often prove difficult to procure in a timely manner” (5). There are currently no medicinal products that have been approved in Europe that are optimized for the intravenous delivery of BCAA-free amino acid supplementation.

In the literature other treatments that have been explored included sodium phenylbutyrate and growth hormone (36, 37). Given the extreme paucity of the literature, neither of these treatments was attempted in the above reported cases.

As conclusion, the use of the IV BCAA-free solution represents an additional treatment modality that may be useful for those patients who are unable to receive oral or enteral BCAA-free amino acid supplementation for reasons such as coma or vomiting and may be particularly useful in a pediatric population where patients present critical episodes and are often unable to tolerate nasogastric feeding. As was highlighted, the ready availability of an IV formulation would represent a welcome solution to an urgent and previously unmet clinical need, enabling prompt effective intervention of metabolic DE in infants and children with MSUD.

## Data availability statement

The raw data supporting the conclusions of this article will be made available by the authors, without undue reservation.

## Ethics statement

The studies involving human participants were reviewed and approved by Ethical Committe Sant Joan de Deu. ID: PIC-131-18. Written informed consent to participate in this study was provided by the participants' legal guardian/next of kin.

## Author contributions

PS-P, ÁG-C, and MC provided data analysis and contributed equally to the preparation and revision of the manuscript. All authors contributed to the article and approved the submitted version.

## Funding

This funding was supported by GAIN. Research group C0-12 IDIS.

## Conflict of interest

The authors declare that the research was conducted in the absence of any commercial or financial relationships that could be construed as a potential conflict of interest.

## Publisher's note

All claims expressed in this article are solely those of the authors and do not necessarily represent those of their affiliated organizations, or those of the publisher, the editors and the reviewers. Any product that may be evaluated in this article, or claim that may be made by its manufacturer, is not guaranteed or endorsed by the publisher.

## Supplementary material

Supplementary Figure 1 Leucine evolution during decompensation episode treatment. Leucine levels are assessed at entrance, end of treatment and discharge.

Supplementary Table 1 IV BCAA-free solution formulation.
