# Supplementary figures and images for: Intravenous branched-chain amino-acid-free solution for the treatment of metabolic decompensation episodes in Spanish pediatric patients with maple syrup urine disease
Source: Front Pediatr. 2022 Aug 15;10:969741. doi: 10.3389/fped.2022.969741 (PMC9420908; doi:10.3389/fped.2022.969741)

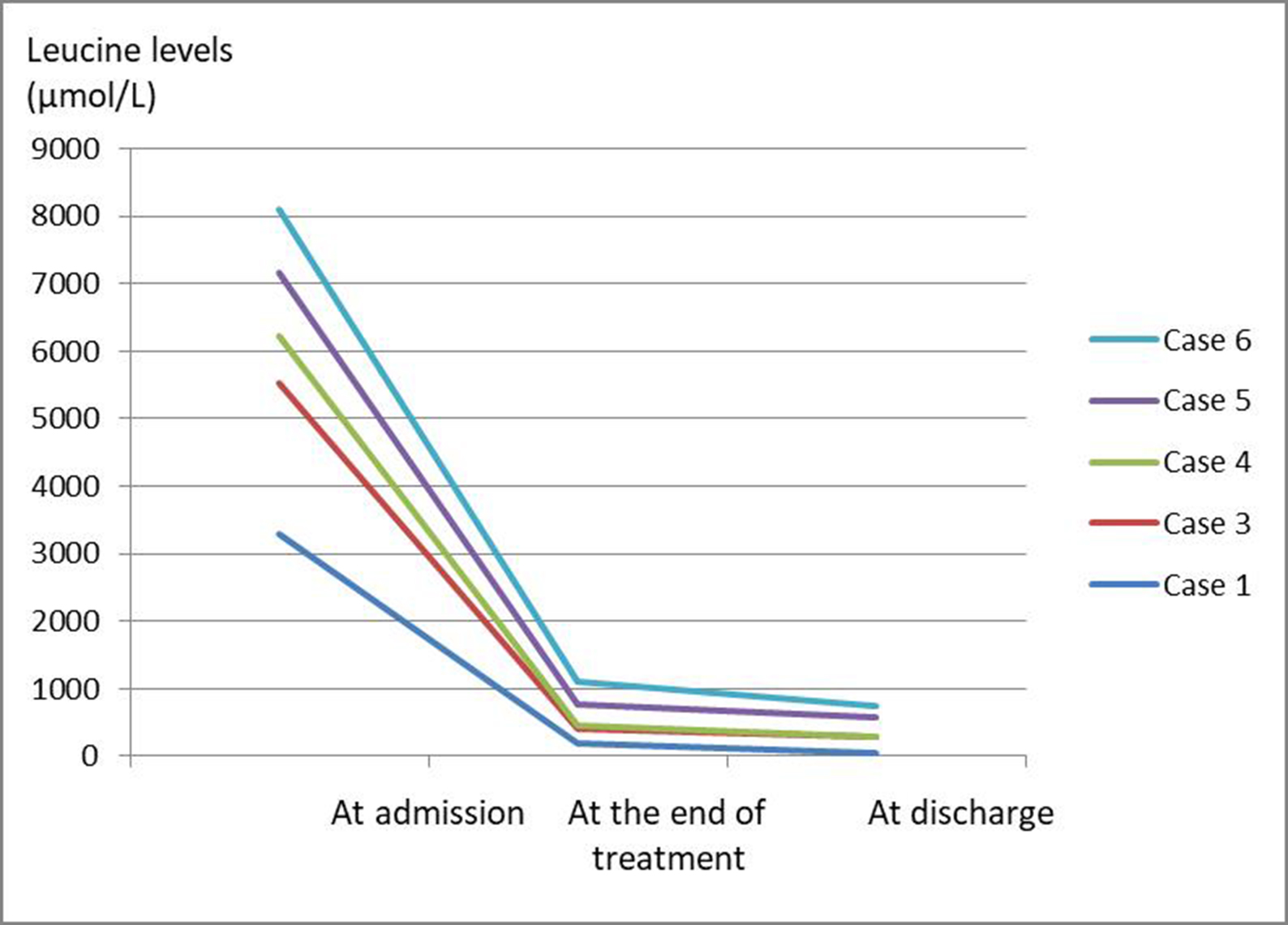

Supplement: Supplementary Figure 1 — Leucine evolution during decompensation episode treatment. Leucine levels are assessed at entrance, end of treatment and discharge. [file Image_1.JPEG]
