# Supplementary material for: Intravenous branched-chain amino-acid-free solution for the treatment of metabolic decompensation episodes in Spanish pediatric patients with maple syrup urine disease
Source: Front Pediatr. 2022 Aug 15;10:969741. doi: 10.3389/fped.2022.969741 (PMC9420908; doi:10.3389/fped.2022.969741)
Supplement: Supplementary Table 1 — IV BCAA-free solution formulation. [file Table_1.DOCX]

Supplementary table 1 – IV BCAA-free solution formulation

| **Ingredients** | **Average content per 1000 mL** |
| --- | --- |
| Alanine | 6.3 g |
| Arginine | 4.1 g |
| Aspartic acid | 4.1 g |
| Cysteine | 1.0 g |
| Glutamic acid | 7.1 g |
| Glycine | 2.1 g |
| Histidine | 2.1 g |
| Lysine | 5.6 g |
| Methionine | 1.3 g |
| Phenylalanine | 2.7 g |
| Proline | 5.6 g |
| Serine | 3.8 g |
| Taurine | 0.3 g |
| Threonine | 3.6 g |
| Tryptophan | 1.4 g |
| Tyrosine | 0.5 g |

*Excipients: acetic acid or sodium hydroxide for pH adjustment, water for injection; amino acid concentration: 52 g/L; pH: 5.2; caloric content: 200 kcal/L and osmolarity: 390 mOsmol/L.*
